# Primary Coronary Embolism as an Unusual Manifestation of Nonbacterial Thrombotic Endocarditis in a Patient with Gastric Cancer

**DOI:** 10.4061/2010/319732

**Published:** 2010-05-31

**Authors:** Giovanni Ferlan, Annalisa Fiorella, Claudio De Pasquale, Francesco Tunzi

**Affiliations:** Department of Cardiac Surgery, University of Bari Medical School, 70122 Bari, Italy

## Abstract

Nonbacterial thrombotic endocarditis (NBTE) is a rare clinical condition characterized by the presence of sterile vegetations on valvular leaflets Gross and Friedberg (1936). The most frequent cause of NBTE is antiphospholipid syndrome Hughson and et al. (1993); malignancy, through an intrinsic condition of hypercoagulability, is the second most common cause Thomas (2001). Systemic thromboembolic complications are frequently associated with this condition, but coronary embolism is not common. We report the case of a patient with NBTE secondary to gastric adenocarcinoma with clinical symptoms of coronary and systemic emboli.

## 1. Case Report

**Figure 1:**
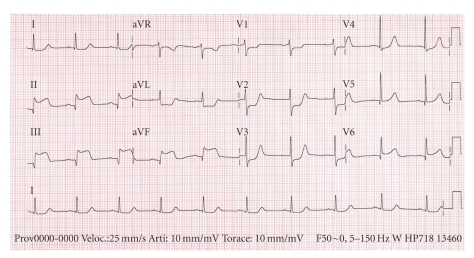
Ecg on admission reveals ST segment elevation in leads II, III, AVF.

**Figure 2:**
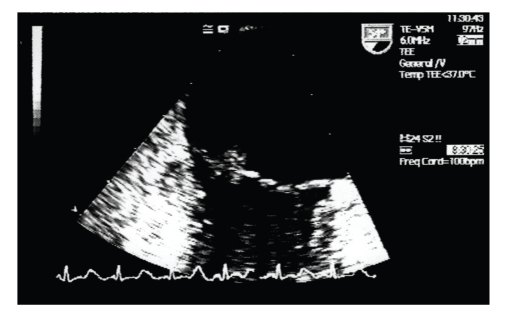
Transesophageal echocardiography shows an echodense structure (0.7 cm by 1 cm) on the atrial aspect of the anterior mitral leaflet.

A 43-year-old previously healthy woman presented with sudden onset of substernal chest pain that had begun some hours earlier. She had no history of coronary artery disease. Physical examination revealed pulse 98 beats/min and blood pressure 95/60 mm, Hg. Electrocardiogram (ECG) showed ST segment elevation in leads II, III, AVF (Figure 1). Myocardial infarction was diagnosed and the patient was admitted to the coronary care unit, where a coronary angiogram demonstrated distal occlusion of the interventricular posterior artery. The following day, the patient developed dysarthria, right-sided weakness, and left homonymous quadrantanopsia. Brain magnetic resonance imaging (MRI) showed several scattered cortical infarcts in the left parieto-occipital region. A transesophageal echocardiogram (TEE), performed to evaluate the neurologic symptoms, revealed on the atrial surface of the mitral valve a 0.7 cm by 1 cm echodense mass on the anterior leaflet (Figure 2) and a 0.6 cm by 0.5 cm vegetation on the posterior leaflet, with evidence of moderate mitral regurgitation. Low-molecular-weight heparin therapy was started. The patient remained afebrile and serial blood cultures were negative. Blood studies revealed moderate thrombocytopenia (50–70.000 per mm3), leukocytosis (20.000 per mm3), and normal hematocrit. Extensive evaluation, including antiphospholipid antibodies, antinuclear antibody, and antineutrophil cytoplasmic antibody, revealed nothing significant. Meanwhile, the patient complained of nausea and persistent vomiting; also an elevated plasma level of CA-19.9 was detected: as a result, a fiberoptic gastroscopy with biopsy was performed. This revealed the presence of a 4 cm by 3 cm mass on the lesser curvature, identified as a gastric adenocarcinoma at the pathological examination. A total-body contrast-enhanced computed tomography showed multiple splenic and renal infarcts but failed to detect any metastatic lesion.

**Figure 3:**
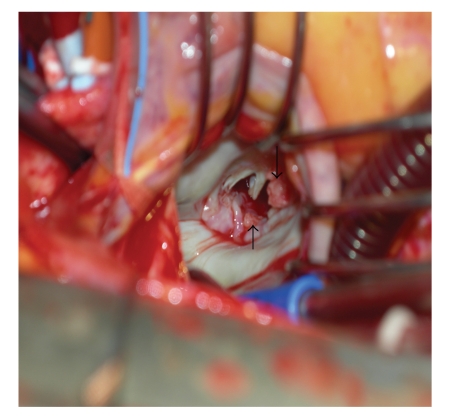
Arrows point to the vegetations on the leaflets.

Consequently, the patient was promptly scheduled for a gastrectomy but suddenly developed a right-sided facial droop and further visual disturbances. A second TEE demonstrated persistence and enlargement of the two vegetations previously seen on the mitral leaflets, in spite of the anticoagulant therapy which had been promptly started. Therefore priority was given to valvular surgery and the patient underwent mitral valve replacement with a bioprostheses without complications. At surgery, the echocardiographic preoperative findings were confirmed (Figure 3). The pathological examination of the excised valve revealed that the vegetations were made up of fibrin and blood platelets; negative stain and cultures confirmed them to be sterile. Granulation tissue and active enlarged fibroblasts were found in the leaflet tissue, at the implant site of the vegetations and all around. On the 10th post-operative day the patient underwent a subtotal gastrectomy and was discharged seven days later. 

## 2. Discussion

 NBTE is characterized by the formation of vegetations on heart valves in the absence of systemic bacterial infections. The vegetations are made up of fibrin and blood platelets and the valvular tissue is either normal or shows some evidence of inflammatory response. As to the pathogenesis, it has been suggested that NBTE could result from local valvular damage mediated by elevated levels of circulating cytokines (such as tumor necrosis factor or interleukin-1). It may also result from the well-known hypercoagulable state associated with malignancies (increased levels of factor VIII, fibrinogen, and von Willebrand factor) [1]. A pre-existing valvular lesion is considered a significant predisposing factor in the pathogenesis of NBTE [1–5]. 

 The incidence of NBTE is uncertain. However, in three large autopsy series published over the last 30 years [6] NBTE has shown an incidence ranging between 1% and 1,6%. In these cases an underlying malignancy was found with a frequency varying between 32% and 80%. On the other hand, an echocardiographic study of 200 living patients with various cancers found evidence of NBTE in approximately 19% [7]. 

 The major clinical manifestations of NBTE result from systemic emboli rather than valvular dysfunction. In the great majority of these patients an embolism occur specially in the spleen, the kidneys, or the central nervous system [6] and can be the main cause of morbidity. In case of coronary embolism, if a pre-existing coronary artery disease has not led to the formation of a collateral coronary circulation, sudden disruption of the blood supply can cause more myocardial damage and a poorer prognosis, and a thrombolytic therapy could therefore seem advisable. However, in case of NBTE, in which sterile valvular vegetations made up of fibrin and platelets are the potential source of coronary embolism, we believe that a thrombolytic therapy, in a similar way as in the patients affected with prosthetic mechanical valve thrombosis, could face a high risk of thromboembolism and ischemic stroke, or to a lesser extent, of major bleeding episodes [8, 9] and must therefore be confined to selected cases.

 Treatment of NBTE includes systemic anticoagulation to prevent recurrent thromboembolism. However, in patients with malignancy-related NBTE, warfarin is often ineffective, probably because of the presence of some nonvitamin K-dependent procoagulant agents which relate to the underlying neoplasm [10] and should not be used. In these cases use of heparin or low-molecular-weight heparin is recommended.

 The indications and appropriate timing of surgical therapy in NBTE have not been formally evaluated. However, in patients with cancers that are potentially curable, the recurrence of embolic events despite anticoagulation and the presence of valvular dysfunction could suggest the need to give priority to the cardiac procedure.

 In the case reported, clinical presentation was unusual: at the onset, embolism first affected coronary circulation, with electrocardiographic and clinical signs of acute inferior myocardial infarction, and later central nervous system. On the basis of the recurrent embolic events registered (despite a correct anticoagulant therapy) and given that the underlying gastric cancer was considered curable, a preliminary valvular surgery seemed advisable.

## References

[1] El-Shami K, Griffiths E, Streiff M (2007). Nonbacterial thrombotic endocarditis in cancer patients: pathogenesis, diagnosis, and treatment. *The Oncologist*.

[2] Gross L, Friedberg CK (1936). Nonbacterial thrombotic endocarditis. Classification and general description. *Archives of Internal Medicine*.

[3] Hughson MD, McCarty GA, Sholer CM, Brumback RA (1993). Thrombotic cerebral arteriopathy in patients with the antiphospholipid syndrome. *Modern Pathology*.

[4] Thomas RH (2001). Hypercoagulability syndromes. *Archives of Internal Medicine*.

[5] Lopez JA, Ross RS, Fishbein MC, Siegel RJ (1987). Nonbacterial thrombotic endocarditis: a review. *American Heart Journal*.

[6] Llenas-García J, Guerra-Vales JM, Montes-Moreno S, López-Ríos F, Castelbón-Fernández FJ, Chimeno-García J (2007). Nonbacterial thrombotic endocarditis: clinicopathologic study of a necropsy series. *Revista Espanola de Cardiologia*.

[7] Edoute Y, Haim N, Rinkevich D, Brenner B, Reisner SA (1997). Cardiac valvular vegetations in cancer patients: a prospective echocardiographic study of 200 patients. *American Journal of Medicine*.

[8] Lengyel M, Fuster V, Keltai M (1997). Guidelines for management of left-sided prosthetic valve thrombosis: a role for thrombolytic therapy. *Journal of the American College of Cardiology*.

[9] Roudaut R, Lafitte S, Roudaut M-F (2003). Fibrinolysis of mechanical prosthetic valve thrombosis: a single-center study of 127 cases. *Journal of the American College of Cardiology*.

[10] Bell WR, Starksen NF, Tong S, Porterfield JK (1985). Trousseau’s syndrome. Devastating coagulopathy in the absence of heparin. *American Journal of Medicine*.

